# 

**Published:** 1908-02-15

**Authors:** 


### BOOKS RECEIVED.

Homœopathic Publishing Co.

"Thomas Skinner, M.D.: A Biographical Sketch." By John H. Clarke, M.D.

"The Enthusiasm of Homœopathy." By John H. Clarke, M.D.

Adam and Charles Black.

"Who's Who?" 1908.

"Who's Who Year-book. 1908.

"The Writers' and Artists' Year-book, 1908."

The J. B. Lippincott Co.

"Abdominal Hernia." By W. B. De Garmo, M.D.

"International Clinics." Vol. IV. 17th series.

Macmillan and Co.

"Primary Nursing Technique." By Isabel McIsaac.

Longmans, Green and Co.

"Proceedings of the Royal Society of Medicine." No. I., Vol. I.

Rebman, Ltd.

"The Master Beast, A.D. 1888-2020: An Imaginative Novel." By Horace W. C. Newte.

"Diseases of the Throat, Nose, and Ear." By D. MacKenzie, M.D.

T. B. Browne, Ltd.

"The Advertiser's A.B.C., 1908."

C. Griffin and Co., Ltd.

"Year-book of the Scientific and Learned Societies, 1907."

### THE GRADUATES' MEDICAL SCHOOLS.

### Diary for the week February 17 to 22.

**THE ROYAL COLLEGE OF SURGEONS.**

**At 5 p.m.**

**Feb. 17, 19, and 21,** Prof. Victor Bonney, **A Study of the Connective Tissues in Carcinoma and in certain pathological conditions preceding its onset.**

**MEDICAL GRADUATES' COLLEGE AND POLYCLINIC, Chenies Street, W.C.**

**Lectures at 5.15 p.m.**

**Feb. 17,** Dr. J. M. H. McLeod, **Lupus Erythematosus.**

**Feb. 18,** Dr. M. C. Hawkes, **Swedish Medical Gymnastics: their Application in the Treatment of Diseases of the Nervous System.**

**Feb. 19,** Dr. J. Mackenzie, **The Nature of the Symptoms in Heart Failure.**

**Feb. 20,** Dr. T. W. Eden, **The Examination of Pregnant Women before Labour.**

**HOSPITAL FOR CONSUMPTION AND DISEASES OF THE CHEST, Brompton, S.W.**

**At 4 p.m.**

**Feb. 19,** Dr. Perkins, **Cases from the Wards.**

**THE HOSPITAL FOR SICK CHILDREN, Great Ormond Street, W.C.**

**At 4 p.m.**

**Feb. 20,** Dr. Still, **Dyspepsia in Children past the Period of Infancy.**

**LONDON SCHOOL OF CLINICAL MEDICINE, Seamen's Hospital, Greenwich.**

**At 2.15 p.m.**

**Feb. 18,** Dr. Hewlett.

**At 3.15 p.m.**

**Feb. 21,** Mr. McGavin, **Cancer of the Œsophagus.**

**THE THROAT HOSPITAL, Golden Square, W.**

**At 5.30 p.m.**

**Feb. 17,** Mr. F. Rose, **Intra-cranial Complications of Ear Diseases.**

**Feb. 20,** Mr. F. Rose, **Intra-cranial Complications: Treatment.**

**NATIONAL HOSPITAL FOR THE PARALYSED AND EPILEPTIC, Queen Square, Bloomsbury, W.C.**

**At 3.30 p.m.**

**Feb. 18,** Dr. Ormerod, **Hysteria.**

**Feb. 21,** Dr. Collier, **Myasthenia Gravis.**

**CHARING CROSS HOSPITAL, W.C.**

Post-Graduate Course.

**At 4 p.m.**

**Feb. 20,** Dr. I. W. Bruce, **Electrical.**

**THE HOSPITAL FOR NERVOUS DISEASES, Welbeck Street, W.**

**At 5 p.m.**

**Feb. 20,** Mr. Laming Evans, **The Use and Abuse of Tendon Transplantation.**

**MOUNT VERNON HOSPITAL, Hampstead.**

Post-Graduate Course.

**At 5 p.m.**

**Feb. 20,** Dr. T. D. Lister, **Demonstration of Cases of Chest Disease** (at the hospital at Hampstead).

**NORTH-EAST LONDON POST-GRADUATE COLLEGE, Prince of Wales' General Hospital, Tottenham, N.**

**At 4.30 p.m.**

**Feb. 20,** Dr. Walter Edmunds, **Surgical Aneurysm.**

**THE POST-GRADUATE COLLEGE, West London Hospital, Hammersmith, W.**

**At noon.**

**Feb. 17,** Dr. Low, **Pathological Demonstration.**

**Feb. 18 and 20,** Dr. Pritchard, **Practical Medicine.**

**At 5 p.m.**

**Feb. 17,** Dr. Davis, **Clinical Lecture II.**

**Feb. 18,** Dr. Moullin, **Gynæcological Cases.**

**Feb. 19,** Dr. Pritchard, **On the Vaccination Treatment of Infective Diseases—I.**

**Feb. 20,** Mr. Edwards, **Clinical Lecture—II.**

**Feb. 21,** Dr. Abraham, **Cases of Skin Disease—II.**

### PLAGUE PREVENTION IN INDIA.

In continuation of his efforts towards combating the scourge of plague, Sir George Clarke, the Governor, recently invited to Bombay Indian medical men from Western India. Several days were spent at the Parel laboratory, where the technical details of anti-plague vaccine manufacture were demonstrated and the visitors were shown how to recognise plague-infected rats and the rat-fleas which convey infection to human beings, together with the best methods of destruction and of house disinfection. At an informal conference Sir George Clarke referred to the value of the lectures delivered by Capt. W. Glen Liston, I.M.S., showing the importance of inoculation. Compulsion was not to be thought of in dealing with the people; the profession should endeavour to carry the masses with them, and by patience and reason induce them to accept the benefits to be derived from inoculation.

### Publications.

*Samples free on application.*

### MEDICAL ACCOUNTS.

Now is the time to arrange for this troublesome business, and
WRIGHT'S THREE-BOOK SYSTEM,
WRIGHT'S SINGLE-BOOK SYSTEM (14th Edition),
WRIGHT'S CHARTS of all kinds (over 670,000 of which have been issued),
WRIGHT'S CHART-HOLDERS (in use all over the world),
WRIGHT'S CERTIFICATE BOOKS, PROFESSIONAL ACCOUNT FORMS, LETTER HEADINGS, DISPENSING LABELS, ETC.,
are most of them better than the ordinary run of these things, and their use saves much time and trouble.

15*th Year. Bound in Rexine, Gilt. Two Pockets, Pencil Case, and Refill.*

### WRIGHT'S IMPROVED VISITING LISTS, 1908

REQUIRE WRITING ONLY ONCE A MONTH.
Compiled by Robert Simpson, L.R.C.P. & S.

| Form A. | s. | d. | | s. | d. |
|---|---|---|---|---|---|
| No. 1.—40 patients | 5 | 0 | No. 4.—160 patients | 5 | 6 |
| No. 2.—80 " | 5 | 0 | No. 5.—200 " | 6 | 6 |
| No. 3.—120 " | 5 | 6 | No. 6.—240 " | 7 | 0 |

Form B (Perpetual List) 5s. 6d. is the same as Form A, but can be commenced at any time and used until filled.

On Sheets 2 ft. 2 in. by 3 ft. 4 in., 2/- each, or 42/- the set of 26 sheets, with Nickeled Head for suspension; or mounted on Linen, 68/-

A SERIES OF

### MIDWIFERY DIAGRAMS:

FOR THE INSTRUCTION OF MIDWIVES AND STUDENTS OF MIDWIFERY.
By VICTOR BONNEY, M.S., M.D., B.Sc. (Lond.), F.R.C.S., M.R.C.P.,
*Lecturer on Practical Midwifery, Middlesex Hospital.*
The figures (160 in number) constitute a complete pictorial course in the subject as far as applicable to those for whom they are designed.
*A full Illustrated Prospectus on application.*

3rd Edition Revised. Pocket Size. 1/- net.

### A GUIDE TO URINE TESTING: For Nurses.

By MARK ROBINSON, L.R.C.P. & S. Edin.
"An excellent little guide—short, concise, and accurate."—*Dub. Med. Jour.*

2nd Edition. 1/-. Pocket Size.

### GOLDEN RULES OF SICK NURSING.

By W. B. DRUMMOND, M.B., C.M., F.R.C.P. Edin.,
*Assist. Phys. at Roy. Hosp. for Sick Children, Edinburgh.*
"From the first page to the last it is full of sound teaching."—*Hospital.*

**Bristol: JOHN WRIGHT & CO. London: SIMPKIN & CO., Ltd.**

### NOTES ON GENERAL PRACTICE.

By S. M. HEBBLETHWAITE, M.D. Lond.

"Sound common sense."—*Hospital.* "We can heartily recommend."—*B. M. Journal.* "A shrewd practical knowledge of everyday general practice."—*Lancet.* "Well worth perusal."—*Scottish Med. & Surg. Journal.* "A great deal of sound advice."—*Edinburgh Med. Journal.* "Will not fail to pleasantly interest."—*Glasgow Med. Journal.* "Well fulfils his hope that they may be found useful."—*Medical Review.* "A very great deal indeed of excellent clinical information."—*Dublin Journal of Med. Sciences.*

Post free 3/9. THE SCIENTIFIC PRESS, 28 Southampton St., W.C.

